# Evaluation of surgical educational videos available for third year medical students

**DOI:** 10.1080/10872981.2020.1714197

**Published:** 2020-01-10

**Authors:** Berina Karic, Veronica Moino, Andrew Nolin, Ashley Andrews, Paul Brisson

**Affiliations:** Department of Surgery, Edward Via College of Osteopathic Medicine, Auburn, AL, USA

**Keywords:** Educational surgery videos, medical education, third year medical student, clinical rotations, laparoscopic appendectomy and laparoscopic cholecystectomy

## Abstract

**Objectives**: In this study we evaluated 40, top recommended, laparoscopic appendectomy and laparoscopic cholecystectomy videos located on public domain websites using eight criteria created by a panel of third year medical students and general surgeons. We hypothesized that there is a lack of quality, thorough educational laparoscopic surgical videos appropriate for third year medical students to review in preparation for the Surgery rotation.

**Methods**: Utilizing a panel, which included four third year medical students and two general surgeons, we created an ‘ideal medical student educational video checklist.’ This checklist included 8 vital criteria. We selected 40, top recommended, videos available on *YouTube* and *Google Video* search engines, using *‘laparoscopic cholecystectomy’* and *‘laparoscopic appendectomy*’ as key terms. Each video was evaluated by four third year medical students individually, using a binary system ‘meets’ or ‘does not meet’ each criterion. Individual scores were averaged, producing a single score for each video.

**Results**: 0/40 (0%) of the videos met all eight of the criteria. 26/40 (65%) of the videos did not meet half of the criteria. The top performing videos 7/40 (17%) only met 5/8 criteria. **Conclusions**: We identified a lack of quality and thorough educational surgical videos appropriate for third year medical students and a need for improved online video based instruction. Our checklist can be utilized as a guide for anyone creating surgical videos for medical student education in the future.

## Introduction

According to recent literature, medical trainees commonly use *YouTube* as their primary preparatory resource for General Surgery cases [1]. The use of multimedia resources, such as surgical videos, requires significantly less study time than classical study preparation, such as reading articles and textbooks [2]. Videos improve clinical reasoning, knowledge, and, by the completion of the surgical rotation, overall evaluation of the student by the preceptor [3]. Friedl *et al*. found that student performance was benefited by multimedia-enhanced teaching compared to print medium, especially in the acquisition of information in temporal and spatial events such as surgeries [2]. There is also evidence suggesting that medical students who watch guided laparoscopic video tutorials score significantly better when tested for anatomical knowledge [4]. However, video-based surgical education located on public domain sites such as *YouTube* have poor quality overall [5]. Thus, the challenge for medical students is to identify the appropriate videos for their level of surgical education.

We were quite disappointed when our initial research identified no standard list of surgical videos recommended for third year medical students or acceptable criteria for the ideal medical student surgical video. That initial experience led us to create a checklist tool to evaluate the most commonly viewed videos. Moreover, this study evaluates the quality of online videos available for the medical trainee. We created an ideal educational video checklist, chose two common general surgery procedures, performed a random online content analysis to select forty top recommended videos, and evaluated those videos using the checklist. We hypothesized that there is a lack of quality, thorough educational laparoscopic surgical videos available for third year medical students to review in preparation for the Surgery rotation.

## Methods

We chose to evaluate videos of two common laparoscopic procedures that third year medical students are likely to encounter during their General Surgery rotation: laparoscopic cholecystectomy and laparoscopic appendectomy.

**Figure 1.**
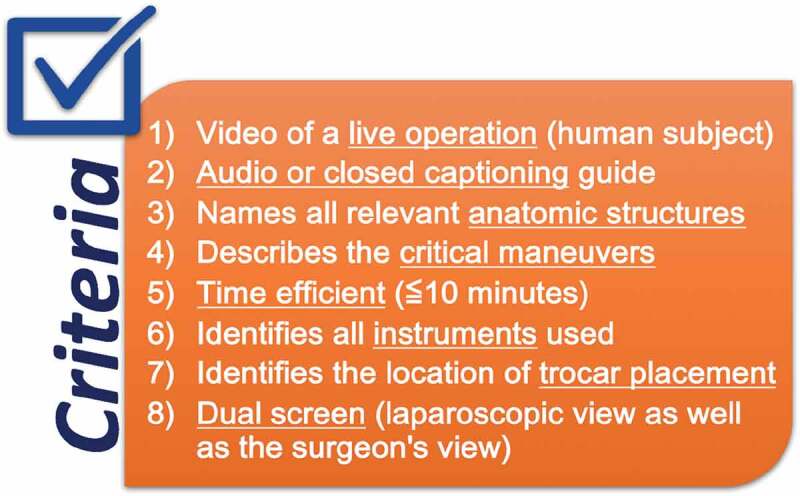
Ideal third year medical student educational video checklist

Prior to viewing the videos, four third year medical students and two general surgeons collaborated to create a checklist of eight criteria that they regarded as essential components of an ideal surgical video for a third year medical student. These essential components were based on a hypothetical ideal video that would show content which a third year medical student would benefit from when viewing a video in preparation for a surgical case. Moreover, the final checklist which we created includes showing a live operation on a human subject, having audio or closed captioning guide throughout the video, naming all relevant anatomic structures as appropriate for the case, describing the critical maneuvers of the procedure, being time efficient, identifying the pertinent instruments used, identifying the location of trocar placement, and showing a dual screen which shows the laparoscopic and surgeon views (Figure 1). These criteria are the most important components for an educational video for third year medical students in training, according to our panel.

We then performed online content analysis to select videos which are freely available on *YouTube* and *Google Video* search engines, using the key words ‘*laparoscopic cholecystectomy*’ and ‘*laparoscopic appendectomy*.’ Our only inclusion criteria was to select videos that showed these procedures. We did not use common abbreviations such as *‘lap chole’* or *‘lap appy,’* or any other abbreviations. Upon retrospective analysis using the listed variations and comparing what is generated in each of the two search engines to our original search queries of ‘*laparoscopic appendectomy*’ and ‘*laparoscopic cholecystectomy*,’ we have found that there was no difference in which videos were generated. The only difference lay in the order in which the videos were listed.

We selected the top recommended videos, which populate first when these key terms are entered, without looking at the number of views each video had. 20 videos, those top recommended by these search engines, were chosen for each laparoscopic procedure, totaling 40 videos (Appendix, Table A1). Each video was individually viewed by four third year medical students who did not have any prior surgical experience, without group discussion.

The videos were graded using a binary system of either meeting (+1) or not meeting (+0) each criterion, giving a total score out of eight for each video. The four individual scores for each video given by the co-investigators were averaged and compiled, giving a single score out of eight for each video. Finally, we calculated the Intraclass Correlation Coefficients (ICC) using the Hertzmark and Spiegelman SAS ICC9 Macro.

## Results

**Figure 2.**
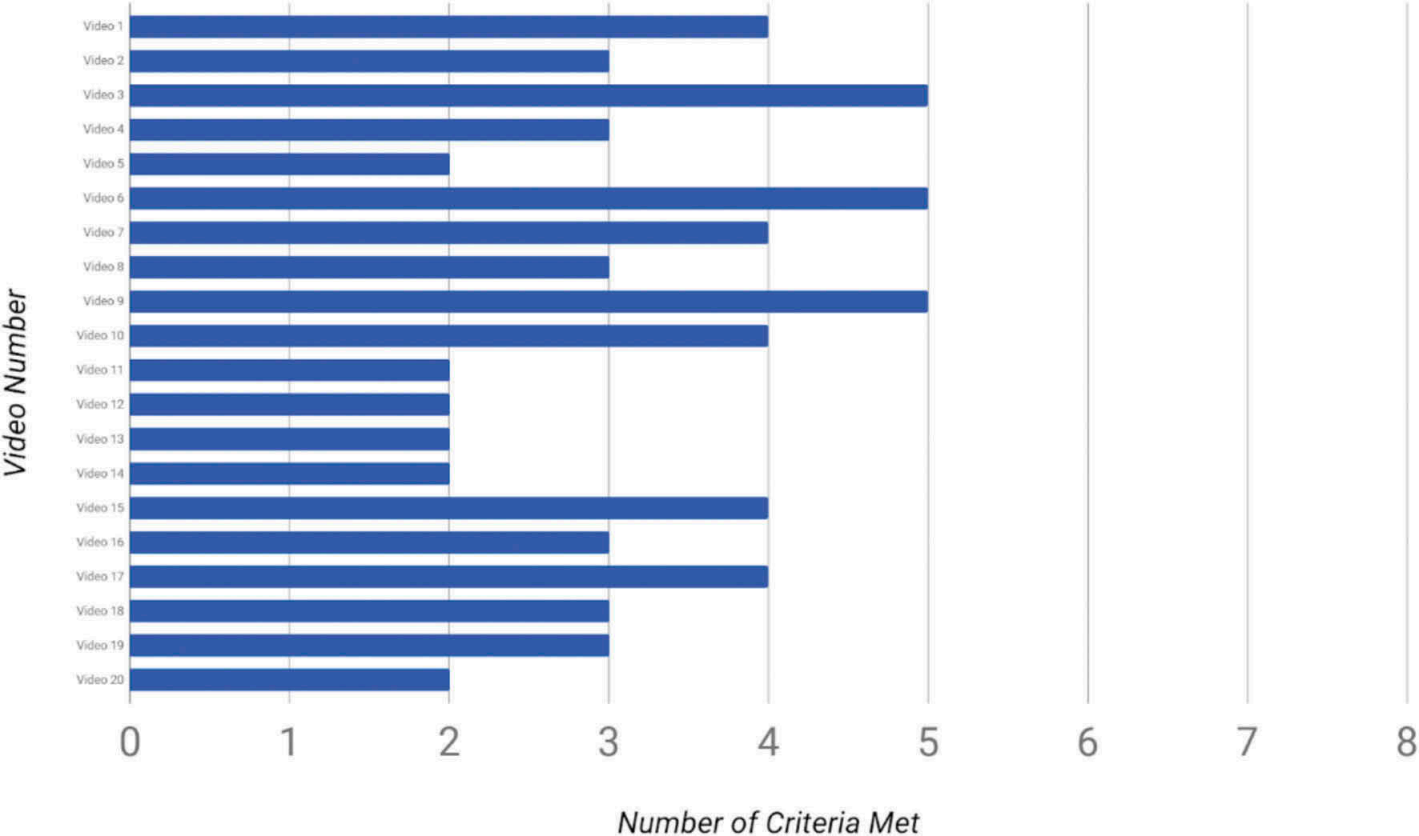
Number of criteria met by each laparoscopic cholecystectomy video

**Figure 3.**
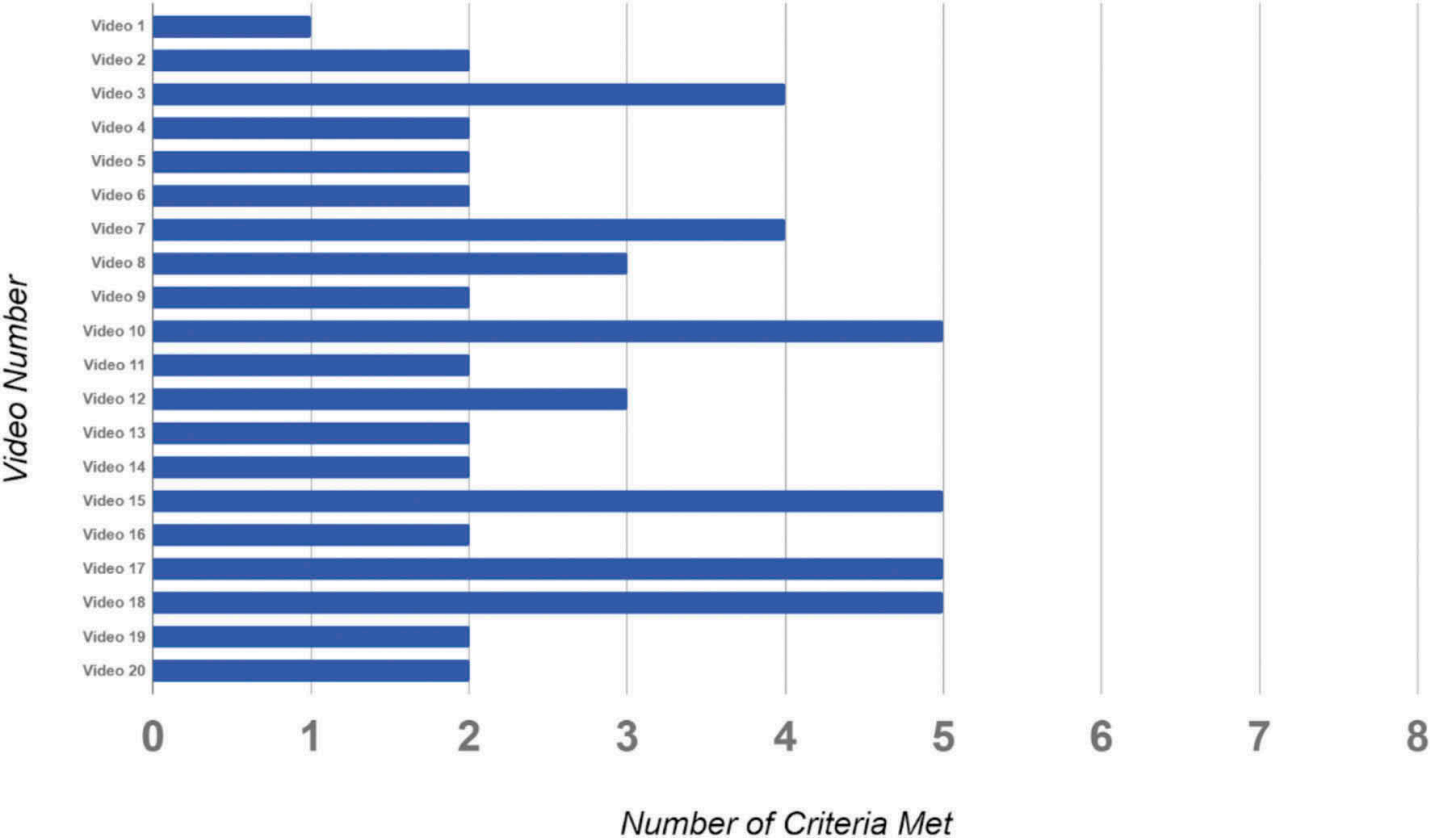
Number of criteria met by each laparoscopic appendectomy video

None, 0/40, of the reviewed laparoscopic cholecystectomy (LC) and laparoscopic appendectomy (LA) videos met all eight criteria from the ideal third year medical student educational video checklist (Figures 2 and 3). The top performing videos, LC number 3, 6, 9 and LA number 10, 15, 17, 18, met 5 of the criteria. These videos are evaluated as ‘best quality’ and can be accessed using the links in Appendix Table A1.

**Figure 4.**
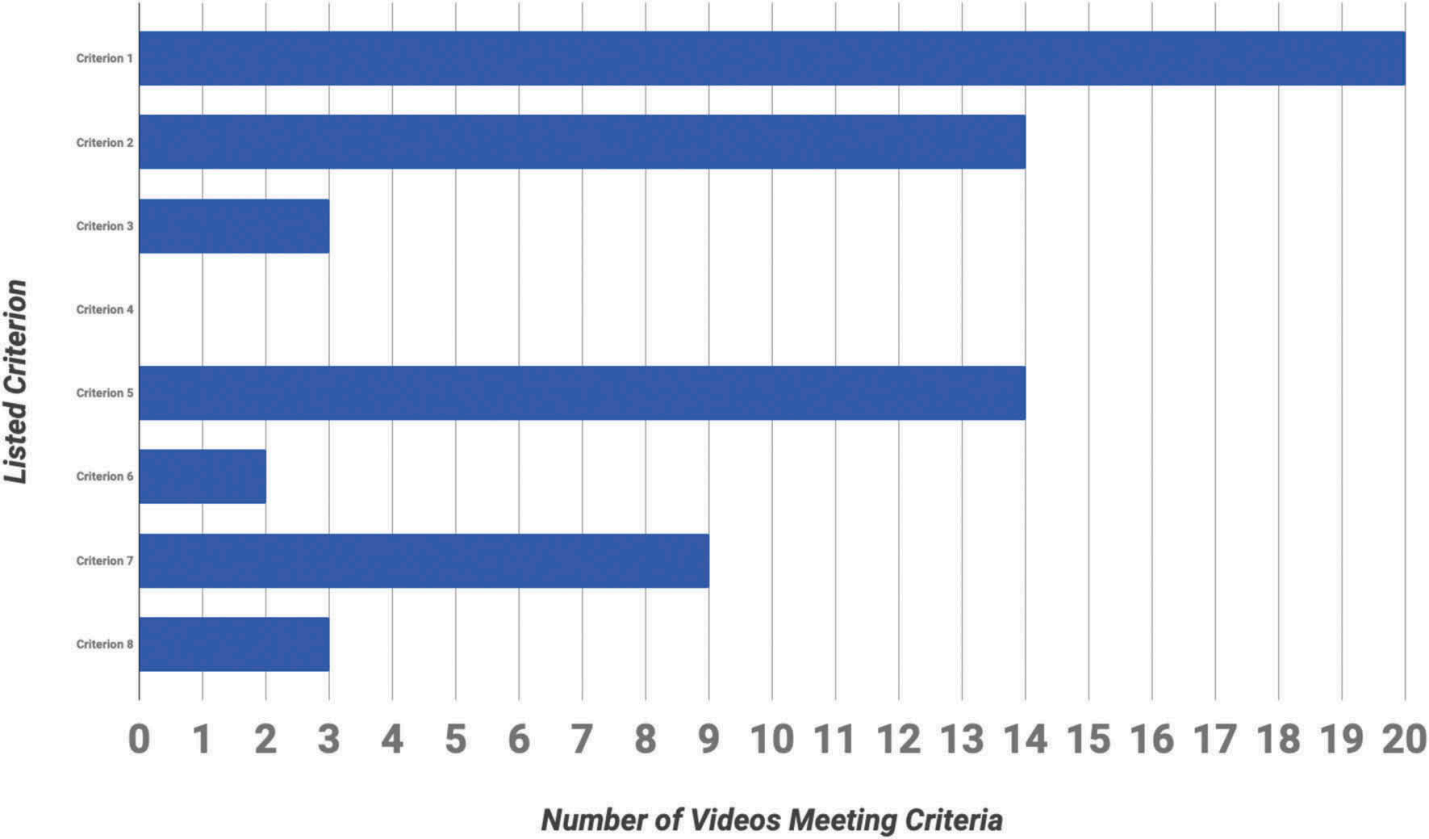
Number of laparoscopic cholecystectomy videos meeting each of the criteria

**Figure 5.**
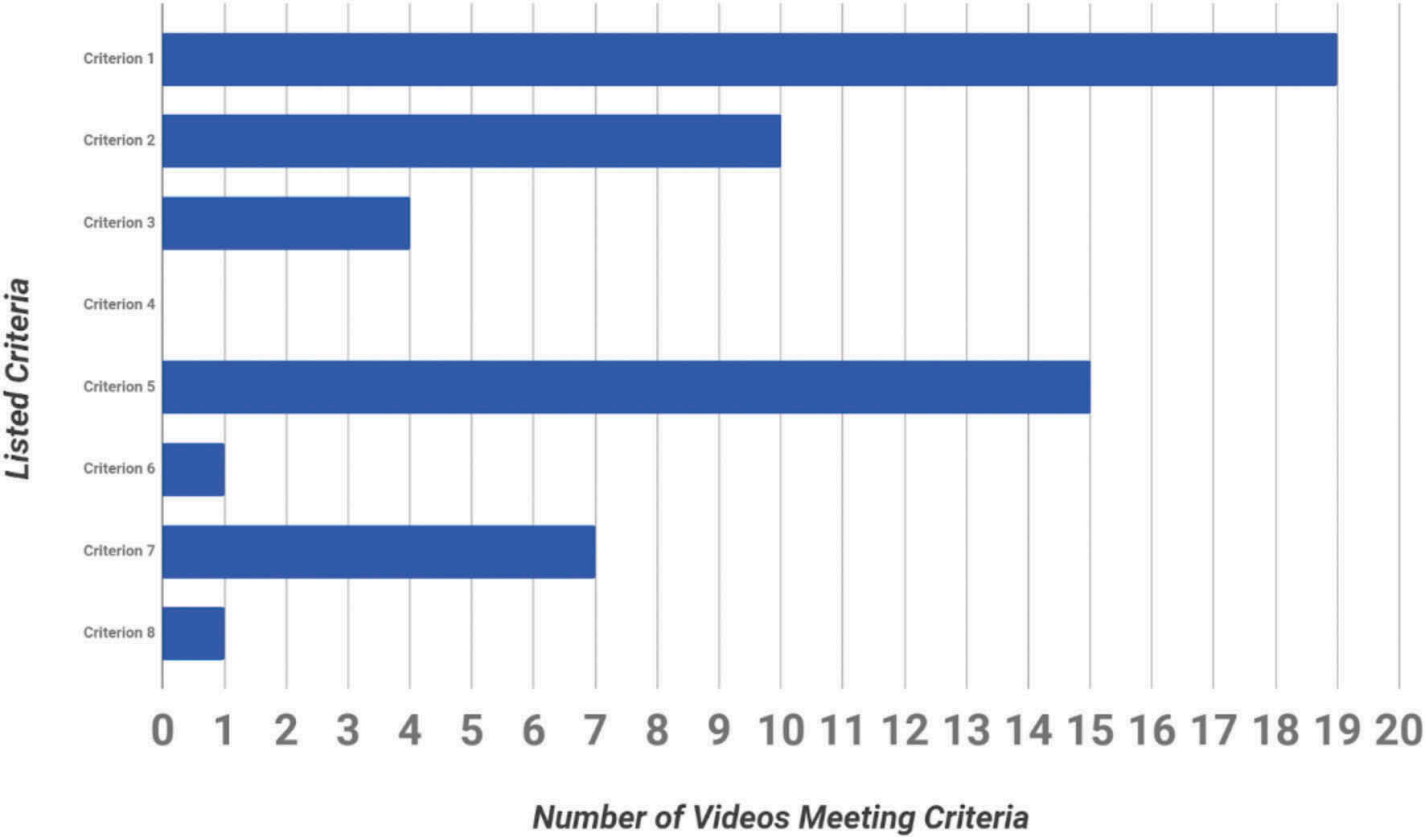
Number of laparoscopic appendectomy videos meeting each of the listed criteria

The most common criterion met by 20/20 (100%) of LC and 19/20 (95%) of LA videos was criterion 1: video of a live operation using a human subject (Figures 4 and 5). None, 0/40, of the videos met criterion 4: describes the critical maneuvers (Figures 4 and 5).

Out of the reviewed videos, 8/20 (40%) LC and 6/20 (30%) LA videos met at least half of the criteria. 60% of LC and 70% of LA videos, overall, 65% (26/40), failed to meet at least half of the criteria.

The Intraclass Correlation Coefficients (ICC) and their 95% confidence intervals, calculated using the Hertzmark and Spiegelman SAS ICC9 Macro, were 0.62 (0.42, 0.79) and 0.60 (0.40, 0.78), for LC and LA respectively.

## Discussion

None of the laparoscopic cholecystectomy or appendectomy videos that we evaluated met all eight criteria from the checklist. More than fifty percent of the videos did not even meet half of our criteria. None of the videos described the critical maneuvers for the specific operation. It is fundamental for educational videos to display and explain the critical or vital steps of the specific operation. Based on these findings, among the vast pool of videos available via common search engines such as *YouTube* and *Google Video*, there exists a possible lack of quality and thorough educational laparoscopic surgical videos for third year medical students to review in preparation for the Surgery rotation.

When viewed in the context of learning styles, the *YouTube* videos may be adequate for the learning style of surgical residents, who already have a developed surgical knowledge base, but may be lacking for the medical students new to the operating room. Engels and de Gara report that medical students have an assimilating learning style, described by Kolb’s learning cycle, in contrast to surgical residents and surgical faculty who have a converging and accommodative learning style [6]. Kolb states that an assimilating learning style prefers ideas and concepts involving a concise and logical approach [7]. Students prefer to watch and think. On the other hand, the converging and accommodating styles prefer a more hands on approach, focused on doing rather than watching [6,7]. The difference in learning styles between medical students and surgical residents and faculty can be explained by differences in base knowledge and experience. Learning through videos offers medical students a concise guide to follow, which resolves the uncertainty from lack of experience and presumptively increases confidence. Future work could include having surgical residents view and grade these videos using our checklist to compare if the available videos are better oriented to trainees with surgical experience.

Surgical training is a lengthy journey; the ‘see one, do one, teach one’ concept, developed by William Stewart Halsted in 1890, is becoming increasingly outdated with the evolution of modern medicine and technologies, leaving junior surgeons less time to learn under real-life conditions [8].

There are a number of limitations in this study. 1) We gave equal weight to each checklist criterion, however, some criteria are arguably more important than the others. A scoring system which weighs each criterion may be more accurate. 2) We choose to evaluate only 40 videos, among the vast number of *YouTube* and *Google* videos available. 3) Our checklist was created based on the opinion of four medical students and two general surgeons. Others may have a different opinion about what criterion should be contained in the checklist. 4) We chose to evaluate recommended views, and we did not take the number of views into account when selecting videos. Future research could include the creation of a scoring system to better evaluate the available videos, evaluation of more videos, and exploring the correlation between the quality of videos and the number of views of each video. Finally, future research could also review the quality of paid video services using our checklist.

## Conclusion

Students rely on educational videos to prepare for their participation in surgical procedures. Our research suggests that there is a great need for educational videos that are appropriate for third year medical students entering their Surgery rotation. Moreover, the ‘ideal medical student video checklist,’ which we developed, can be used as a guide for creating quality surgical educational videos in the future.

## APPENDIX

**Table A1.** Top recommended LC and LA videos

	Laparoscopic Cholecystectomy	Laparoscopic Appendectomy
**1.**	**https://www.youtube.com/watch?v=rXBUXmqc3ro**	**https://www.youtube.com/watch?v=VfT–J-MZlM**
**2.**	**https://www.youtube.com/watch?v=n18zxNGJdLE**	**https://www.youtube.com/watch?v=R9w_6F4hzD0**
**3.**	**https://www.youtube.com/watch?v=YOAkvU8Uxhw**	**https://www.youtube.com/watch?v=opb4dUw_ZZ8**
**4.**	**https://www.youtube.com/watch?v=DrEt3uDTC04**	**https://www.youtube.com/watch?v=4s69S1u5YdA**
**5.**	**https://www.youtube.com/watch?v=VwYfcJzFZaY**	**https://www.youtube.com/watch?v=73jhy1bi2MY**
**6.**	**http://www.anatomyguy.com/laparoscopic-cholecystectomy/**	**https://www.youtube.com/watch?v=E1ljClS0DhM**
**7.**	**https://www.youtube.com/watch?v=a7rIFlvZM0I**	**https://www.youtube.com/watch?v=M2_VvTc2nOg**
**8.**	**https://www.youtube.com/watch?v=Fsp9Rk0laVA**	**https://www.youtube.com/watch?v=AwRCrcifI70**
**9.**	**https://www.youtube.com/watch?v=fz48sITkdJ0**	**https://www.youtube.com/watch?v=FmtsCHUri2c**
**10.**	**https://www.youtube.com/watch?v=rPuYHj1IDKc**	**https://www.youtube.com/watch?v=cMB5qm6YkBE**
**11.**	**https://www.youtube.com/watch?v=PtAdtXWBG6o**	**https://www.youtube.com/watch?v=x8sUeH5M5Q0**
**12.**	**https://www.youtube.com/watch?v=VC4UebchBIQ**	**https://www.youtube.com/watch?v=AD1TM9kf7ak**
**13.**	**https://www.youtube.com/watch?v=mARLWOggrFQ**	**https://www.youtube.com/watch?v=4yo_MF9aZHE**
**14.**	**https://www.youtube.com/watch?v=b2P-w57RXUE**	**https://www.youtube.com/watch?v=vJT09sJKcM4**
**15.**	**https://msurgery.ie/laparoscopic-cholecystectomy**	**https://msurgery.ie/proc1-lapapp**
**16.**	**https://www.youtube.com/watch?v=xoN02albOjY**	**https://reference.medscape.com/viewarticle/827352**
**17.**	**https://www.youtube.com/watch?v=Y45sMvxx8Ag**	**https://www.youtube.com/watch?v=18eYVp244mQ**
**18.**	**https://www.youtube.com/watch?v=RgcECkFJrUc**	**https://www.youtube.com/watch?v=mbk36SrBkfU**
**19.**	**http://pie.med.utoronto.ca/TVASurg/project/standard_lap_cholecystectomy/**	**https://www.youtube.com/watch?v=J7IbZmqhVvU**
**20.**	**https://www.youtube.com/watch?v=FQarwJ4Y7Sw**	**https://www.youtube.com/watch?v=7TONn2-swBc**
